# HIV prevalence and behavioral studies in female sex workers in Togo: a decline in the prevalence between 2005 and 2011

**DOI:** 10.11604/pamj.2013.15.62.2457

**Published:** 2013-06-21

**Authors:** Palokinam Pitché, Komi Gbetoglo, Bayaki Saka, Séfako Akakpo, Dadja Essoya Landoh, Stéphane d'Alméida, Abiba Kere Banla, Dométo Sodji, Kodzo Deku

**Affiliations:** 1 Ministry of Health, Togo; 2 Department of Dermatology and Sexually Transmitted Infections (STIs), CHU Sylvanus Olympio, University of Lomé, Togo; 3 Demographic Research Unit, University of Lomé, Togo; 4 NGOs FAMME, Lomé, Togo

**Keywords:** Sex workers, HIV, sexual behavior, Togo

## Abstract

**Introduction:**

We determined the sero-prevalence of HIV among female sex workers (FSWs) in Togo identified their sexual risk behaviors.

**Methods:**

We conducted a cross-sectional study from 17 to 27 December, 2011 on 1106 FSWs in Togo. Venous sample were collected to estimate HIV prevalence as per national algorithms. Behavior data were collected by interviewer-administered questionnaires.

**Results:**

Of the 1106 FSWs (mean age = 27.6 years) surveyed, 17% and 63% had their first sexual intercourse before the age of 15 and 18 years respectively. Overall, 43.4% of the FSWs had more than seven clients per week. Most FSWs (95%) said they had sex using a condom in their lifetime while 8.8% had used a condom during their last sexual intercourse. About 79% of FSWs used a condom during their sexual encounters the previous week and 11.6% had used a condom during each of their sexual encounters the previous day. Most FSWs (62.2%) reported to have been tested for HIV. Of these, 145 (13.1%) were HIV positive. HIV sero-prevalence decreased from 19.4% in the south to 7.5% in the north of the country. Behaviors associated with FSW being HIV positive included: FSW having more than 7 clients per week (p < 0.001), not using condoms at every intercourse act (p = 0.003) or during the last sexual encounter (p = 0.006) and trading sex in brothels (p < 0.001).

**Conclusion:**

We estimate HIV sero-prevalence among FSWs in 2011 to be 13.1% in Togo, significantly lower than a prevalence of 29.5% estimated previously in 2005. Inconsistent use of condoms was identified as associated with high risk factor for acquiring HIV.

## Introduction

By the end of 2011, UNAIDS estimated that the number of People Living With HIV/AIDS (PLWHA) worldwide was 34 million, of which 69% live within sub-Saharan Africa [1]. An estimated 2.5 million new infections occurred in 2011, representing a decrease of 20% compared to 2001 [1]. Over a period of ten years, this decline is notable in Sub-Saharan Africa, where there are a significant number of countries that have seen a reduction by more than 25% [1].

Despite this, Sub-Saharan Africa recorded 71% of all new infections in 2011 in the world. Female sex workers (FSWs) are one of the most important groups propelling the epidemic in most countries in Africa [1, 2]. Indeed, even when prevalence rates are generally quite low in a country, they can be very high in this group. HIV prevalence among sex workers is 13.5 times higher than among other women [2].

In Togo, the second generation surveillance conducted by the National AIDS and STI Program (NAP) estimated HIV prevalence in FSWs in the city of Lomé to be 54.7% in 2003; 11 times the prevalence estimated for the general population [3]. To address the problem, a concerted effort was carried out by various actors in the field of prevention of HIV infection between 2003 and 2005 to. In 2005, there was a shift in HIV prevalence among FSWs, declining to 45.4% in Lomé and to 29.5% nationally [3].

Taking into account the importance of FSWs in the dynamics of the epidemic in Togo, specific prevention programs targeting this population have been implemented. Five years after the last survey, a new study was necessary in order to measure changes in the prevalence of HIV among these FSWs and to adjust our strategy for the coming years.

## Methods

### Study population and sampling

This study targeted female sex workers (FSWs). Male prostitution is identified as very limited in Togo. FSW was defined as any female who exchanges sexual favors for a fee, occurring in brothels (brothel-based FSWs), hotspots (street-based and bar-based FSWs) or covert FSWs (private sex workers or client contacts sex worker by phone or via hotel staff).

In the strategy of support, the Togolese National AIDS/STDs Control Programme (PNSL-IST) distinguishes two groups of FSWs: street-based and/or covert FSWs and brothel-based FSWs who work almost exclusively in brothels.

Togo is a West African country, which had 6 million inhabitants in 2010. A national map of Sex workers was developed in 2009 determining the number of FSW per region and identifying sites of prostitution [4]. This study enumerated 8,000 FSWs, including 490 brothel-based FSWs, 1548 street-based FSWs and 5962 covert FSWs.

These results were used as a sampling frame for the subgroups of FSWs in each prefecture of the country and in the health districts of Lomé. A sample size of 1300 FSWs was calculated. Based on this, it was decided to interview all FSWs operating in brothels due to the convenience of accessing this population group as compared to other categories of FSWs. Sampling was, however, also performed among bar-based and street-based FSWs in proportion to the weight of each category.

### Data collection

The study was carried out using the established protocol (the second-generation survey generic protocol developed by WHO and UNAIDS) and validated by the national reference group for monitoring and evaluation and research of the Togo National AIDS and sexually transmitted infections control Council (CNLS-IST).

The survey team was trained on both behavioral and serological aspects of the protocol. After training, the data collection tools where pretested in two non-participating areas in order to improve on their efficiency and accuracy. Data collection for the study took place from December 17 to 27, 2011 throughout the national territory.

The data collection on the behavioral component used an individual questionnaire with closed questions. The respondents were then subjected to a blood test to measure the prevalence of HIV (testing from venous blood following the tests prescribed by the national algorithm).

### Data processing and analysis

Completed questionnaires were checked for errors before being computerized. The collected data were entered by trained officers to record information using the software CSPro4.0. The results biological tests were also entered in the database. Data collected were cleaned and analyzed using SPSS software. Descriptive analyses were performed using Chi-square test at a significance level of 5%.

### Ethical considerations

The entire study protocol (including questionnaires and consent forms; Ref No: 0176/2011/MS/CAB/DGS/DPLET/CBRS) were approved by the board committee of the Bioethics Committee of the Ministry of Health. We obtained consent from study subjects that participated in the study. For each of the FSWs surveyed, the objectives, benefits to participate in the survey and progress of the investigation were clearly stated as well as their right to interrupt the interview without justification. An informed consent form signed after the verbal explanation was made by the investigating officer in the language understood by the FSW.

The survey was “anonymous” and confidential. The respondents’ names were not recorded to insure their confidentiality; these were replaced by numbers. Sample collection was conducted by laboratory technicians and medical personnel from district and regional health facilities under the supervision of pathologists from the National Institute of Hygiene (INH).

## Results

### Socio-demographic characteristics of FSWs

In total, 1106 FSWs, were surveyed; the response rate was 85%. Of these, 73.4% were covert and street based. The average age was 27.6 years (range: 14 years and 68 years). Most (73.5%) were 18 and 34 years and only 5.6% of them were younger than 18 years. The majority of FSWs was not in a permanent relationship (66.9%) and had a primary and secondary education level (65.2%). Approximately half of the FSWs (41%) resided in Lomé, the capital of the country. FSWs operated mainly in bars (51%) and in brothels (26%).

### Sexual behavior and HIV testing

Approximately 17% of FSWs had their first sexual intercourse before the age of 15 years. Before the age of 18 years this proportion increased to 63%. Also, 56.6% of FSWs had less than 7 clients a week. Sexual activity was earlier in younger generations than in older generations. Thus, 59.7% of FSWs under the age of 18 years, whereas 7% of FSW aged 40 and over had their first sexual intercourse before the age of 15 years. In addition, 71.2% of FSWs under the age of 25 years had their first sexual intercourse before the age of 18 years against 44.4% of those aged 40 and above.

For all FSWs, the average number of clients seen per week was 37 (range 1 to 52). A statistically significant difference between the average number of clients seen per week existed between brothel-based FSWs (40 clients) and combined covert and street-based FSWs (36 clients) (p = 0.0002).

According to the FSWs, 95% said they have had sex using a condom at least once in their lifetime, 8.8% said they had used a condom during their last sexual intercourse. Only 11.6% used condoms during their last sexual encounter lower than that of FSWs who have used a condom during their lifetime (p <0.001). Moreover, 79% of FSWs used a condom during the last week of work. The main reason for condom use mentioned by FSWs was to prevent HIV/AIDS (96%), STI (78%) and unwanted pregnancies (54%). About one third (34%) of FSWs who had used lubricants to facilitate sex. Finally, 688/1106 FSWs (62.2%) reported having been tested for HIV; of them 93% declared to have collected their results.

### HIV sero-prevalence

Of the 1106 FSWs, 145 (13.1%) were identified as being HIV positive by serology test. The HIV sero-prevalence was 21.8% among brothel-based FSWs against 10% in covert and street based FSWs (p < 0.001). Approximately one in four FSWs (24.4%) from the age of 35 to 39 years were HIV positive, followed by 22% of FSWs from 30 to 34 years. HIV sero-prevalence was 19.4% in the region of Lomé and decreased from south to north of the country (Table 1). Four types of behavior were associated with HIV sero-prevalence among FSWs including: having over 7 clients per week (p <0.001), no-use of condoms during each sex (p = 0.003), non-use of condoms during last sex (p = 0.006) and open sex trade by brothel-based FSWs (p =0.001) (Table 2).

**Table 1:** HIV sero-prevalence among FSWs by socio-demographic characteristics, Togo, 2011

Features	HIV Negative n (%)	HIV Positive n (%)
Type of FSW*		
Brothel-based	230 (78.2)	64 (21.8)
Street-based and covert	730 (90.0)	82 (10.0)
**Age (years)**		
>18	61 (98.4)	1 (1.6)
18-24	404 (91.0)	40 (9.0)
25-29	213 (88.4)	28 (11.6)
30-34	99 (78.0)	28 (22.0)
35-39	68 (75.4)	22 (24.4)
≥40	116 (81.7)	26 (18.3)
**Education level**		
Non schooled	241 (84.0)	46 (16.0)
Primary	300 (84.7)	54 (15.3)
Secondary	328 (89.4)	39 (10.6)
High school and above	92 (93.9)	6 (6.1)
**Health regions**		
Lomé commune	365 (80.6)	88 (19.4)
Maritime	114 (89.8)	13 (10.2)
Plateaux	189 (92.6)	15 (7.4)
Centrale	32 (82.1)	7 (17.9)
Kara	83 (92.2)	7 (7.8)
Savanes	178 (92.2)	15 (7.8)

* FSW = Female sex workers

**Table 2:** HIV sero-prevalence among FSWs by sexual behavior, Togo, 2011

	HIV Negative n (%)	HIV Positive n (%)	p value
**Age at first intercourse**			
No sex before age 18	348 (85.1)	61 (14.9)	0.173
Had sex before age 18	613 (87.9)	84 (12.1)	
**Number of clients per week**			
Less than seven clients	563 (89.9)	63 (10.1)	< 0.001
Seven clients or more	398 (82.9)	82 (17.1)	
**Use of condoms at every act of intercourse during the last working day**			
No	839 (85.8)	139(14.2)	0.003
Yes	122 (95.3)	6 (4.7)	
**Frequency of listening to messages**			
Frequent exposure to key messages	192 (88.5)	25 (11.5)	0.996
Bystander	446 (88.5)	58 (11.5)	
**Precautionary prevention of STI/HIV/AIDS**			
No precautions	61 (92.4)	5 (7.6)	0.17
Condom use	900 (86.5)	140(13.5)	
**Condom use at last sex**			
No	868 (86.0)	141(14.0)	0.006
Yes	93 (95.9)	4 (4.1)	
**HIV test**			
Yes	589 (85.6)	99 (14.4)	0.137
No	371 (88.8)	47 (11.2)	
Type of FSW*			
Brothel-based	230 (78.2)	64 (21.8)	< 0.001
Street-based and covert	730 (90.0)	82 (10.0)	

* FSW = female sex workers

## Discussion

This study has documented HIV sero-prevalence and behaviors of FSWs in 2011. It is a follow up of the study conducted in the same group in 2005. In six years, we have noticed a decline in HIV prevalence from 29.5% to 13.1%, a decrease of 56%. This drop was observed in all health regions of the country (Table 3). The survey also confirmed that FSWs in Togo are concentrated in the capital, Lomé (41% of surveyed FSWs). In 2005, 52% of the 5397 FSWs identified in Togo lived in Lomé, where only 15% of the Togolese population lived [3]. These results suggest the positive effect of the national strategy established by the National Council to fight against HIV/AIDS which has targeted this population by offering services tailored to FSWs since 2005.

**Table 3:** Changes in HIV sero-prevalence among FSW between 2005 and 2011, Togo

Regions	2010 [3]			2011			Rate of decline (%)
	FSWs* HIV positive (n)	FSWs* tested (n)	Prevalence (%)	FSWs* HIV positive (n)	FSWs* tested (n)	Prevalence (%)	
Lomé commune	163	359	45.4	88	453	19.4	57
Maritime	26	66	39.4	13	127	10.2	74
Plateaux	21	118	17.8	15	204	7.4	58
Centrale	19	100	19	7	39	17.9	6
Kara	19	137	13.9	7	90	7.8	44
Savanes	15	145	10.3	15	193	7.8	24
Total	273	925	29.5	145	1106	13.1	56

* FSW = Female sex workers

With regards to the use of condoms, 95% of FSWs reported that they have had sex with a condom at least once in their lifetime. This rate is close to the 97% of FSW who reported condom use at least once in their lifetime in Nigeria [5]. Only 11.6% of FSWs have used condoms during their last sexual encounter; this indicates that 88.4% of FSWs were not protected during their latest intercourse. In our study, 8.8% of FSWs said they had used a condom during their last sexual intercourse. This proportion is substantially lower than that 85% of FSW who reported using a condom during their last sexual intercourse reported by UNAIDS from a survey of capital cities in 85 countries [1]. Likewise, a study in Rwanda showed that 74% of FSWs had used a condom during their last sexual intercourse [6]. In our study, 79% of FSWs used a condom during sexual intercourse with clients in the last week of work. In Uganda, 94% of FSWs reported having used a condom during the previous month of work, and among them, 45% had consistently used it [7]. These results show that the use of condoms by FSWs must be improved if we want to significantly reduce the prevalence of HIV with lasting effect, and attain the Millennium Development Goals by 2015.

In our study, 62.2% of FSWs reported having been tested for HIV, and among them, 93% said they collected their results, implying they knew their HIV status. In Guinea, all FSWs reported having carried out their HIV test, and 92% among them collected their results [8]. Continued awareness campaigns for FSWs by peer educators should be encouraged to explain the importance of screening test and encourage them to know their HIV status.

We measured a HIV prevalence of 13.1% amongst FSWs. This prevalence is four times higher than that of the general population (3.2%), Previous estimates of HIV prevalence among FSWs in sub-Saharan Africa countries ranges from 24 to 46.5% [6, 9–14]. In a review by Baral et al. [2] the average HIV prevalence among FSWs in 50 countries was estimated at 12%. In 2005, the HIV prevalence among FSWs in Togo was 29.5% [3]; suggesting a decrease of 56% between 2005 and 2011 when compared to the estimate in our study. These results are encouraging. Indeed, Togo is one of the 25 countries in the world where incidence of new HIV infections dropped by over 50% between 2001 and 2011 [1]. Although a remarkable reduction has been observed across Togo, HIV prevalence remains high in the country's capital when compared to other geographic health regions.

This study had limitations related to the methodology used. FSWs are difficult to locate due to stigma as well as their illegal status in Togo. We therefore used convenient sampling methods to select the study participants. We did not have information on non-respondents and refuters; but our study had a response rate of 85%. Also, a previous map of sex workers was used to identify brothels and hotspots. Finally, data collection was based on face-to-face administrated questionnaires which method is subject to information bias arising from concealment of information.

## Conclusion

Sexual behaviors identified in this survey (i.e. number of clients per week, consistency in use of condoms and openly trading sex) reflect a cumulative risk of exposure to HIV infection. These factors were taken into account by the CNLS-IST under the national strategy against AIDS 2012-2015. Indeed, it is by addressing the determinants of the epidemic, the specific needs of this population, increasing the supply of services of prevention and providing care tailored to each target population, that we can hope to sustainably impact on the HIV epidemic throughout Africa.

## References

[1] UNAIDS (2012). Report on the global AIDS epidemic.

[2] Baral S, Beyrer C, Muessig K, Poteat T, Wirtz AL, Decker MR (2012). Burden of HIV among female sex workers in low-income and middle-income countries: a systematic review and meta-analysis. Lancet Infect Dis..

[3] Sobéla F, Pépin J, Gbéléou S, Banla AK, Pitché VP, Adom W (2009). A tale of two countries: HIV among core groups in Togo. J Acquir Immune Defic Syndr..

[4] Conseil national de lutte contre le SIDA et les IST (CNLS-IST) (2009). Mapping des principaux sites de prostitution des six régions sanitaires du Togo. http://www.cnlstogo.org.

[5] Onyeneho NG (2009). HIV/AIDS risk factors and economic empowerment needs of female sex workers in Enugu Urban, Nigeria. Tanzan J Health Res..

[6] Braunstein SL, Ingabire CM, Geubbels E, Vyankandondera J, Umulisa MM, Gahiro E (2011). High burden of prevaent and recently acquired HIV among female sex workers and female HIV voluntary testing center clients in Kigali, Rwanda. PLoS One..

[7] Matowu JK, Ssebadduka BN (2012). Sexual risk behaviours, condom use and sexually transmitted infection treatment-seeking behaviours among female sex workers and truck drivers in Uganda. Int J STD AIDS..

[8] Aho J, Nguyen VK, Diakité S, Sow A, Koushik A, Rashed S (2012). High acceptability of HIV voluntary counseling and testins among female sex workers: impact of individual and social factors. HIV Med..

[9] Lawan UM, Abubakar S, Ahmed A (2012). Risk perceptions, prevention and treatment seeking for sexually transmitted infections and HIV/AIDS among female sex workers in Kano, Nigeria. Afr J Reprod Health..

[10] Mbonye M, Nalukenge W, Nakamanya S, Nalusiba B, King R, Vandepitte J (2012). Gender inequity in the lives of women involved in sex work in Kampala, Uganda. J Int AIDS Soc..

[11] Vuylsteke B, Semdé G, Sika L, Crucitti T, Ettiègne Traoré V, Buvé A (2012). HIV and STI prevalence among female sex workers in Côte d'Ivoire: why targeted prevention programms should be continued and strengthened. PLoS One..

[12] Forbi JC, Entonu PE, Mwangi LO, Agwale SM (2011). Estimates of human immunodeficiency virus incidence among female sexe workers in North central Nigeria: implications for HIV clinical trials. Trans R Soc Trop Med Hyg..

[13] Braunstein SL, Umulisa MM, Veldhuijzen NJ, Kestelyn E, Ingabire CM, Nyinawabega J (2011). HIV diagnosis, linkage to HIV care, and HIV risk behaviors among newly diagnosed HIV-positive female sex workers in Kigali, Rwanda. J Acquire Immune Defic Syndr..

[14] Ahoyo AB, Alary M, Ndour M, Labbé AC, Ahoussinou C (2009). HIV and sexually transmitted disease among sex workers in Benin. Med Trop..

